# The Role of Protein Crystallography in Defining the Mechanisms of Biogenesis and Catalysis in Copper Amine Oxidase

**DOI:** 10.3390/ijms13055375

**Published:** 2012-05-03

**Authors:** Valerie J. Klema, Carrie M. Wilmot

**Affiliations:** Department of Biochemistry, Molecular Biology, and Biophysics, University of Minnesota, 6-155 Jackson Hall, 321 Church St. SE, Minneapolis, MN 55455, USA; E-Mail: klema007@umn.edu

**Keywords:** amine oxidase, copper, biogenesis, catalysis, cofactor, protein crystallography

## Abstract

Copper amine oxidases (CAOs) are a ubiquitous group of enzymes that catalyze the conversion of primary amines to aldehydes coupled to the reduction of O_2_ to H_2_O_2_. These enzymes utilize a wide range of substrates from methylamine to polypeptides. Changes in CAO activity are correlated with a variety of human diseases, including diabetes mellitus, Alzheimer’s disease, and inflammatory disorders. CAOs contain a cofactor, 2,4,5-trihydroxyphenylalanine quinone (TPQ), that is required for catalytic activity and synthesized through the post-translational modification of a tyrosine residue within the CAO polypeptide. TPQ generation is a self-processing event only requiring the addition of oxygen and Cu(II) to the apoCAO. Thus, the CAO active site supports two very different reactions: TPQ synthesis, and the two electron oxidation of primary amines. Crystal structures are available from bacterial through to human sources, and have given insight into substrate preference, stereospecificity, and structural changes during biogenesis and catalysis. In particular both these processes have been studied *in crystallo* through the addition of native substrates. These latter studies enable intermediates during physiological turnover to be directly visualized, and demonstrate the power of this relatively recent development in protein crystallography.

## 1. Introduction

Amine oxidases are responsible for the oxidative deamination of amines to their corresponding aldehydes sequentially coupled to the reduction of O_2_ to H_2_O_2_. Members of this ubiquitous group of enzymes can be further classified based on the chemical identity of the organic cofactor used during catalysis, into either the flavin adenine dinucleotide-containing amine oxidases (monoamine oxidase (MAO) (E.C. 1.4.3.4) and polyamine oxidase (E.C. 1.5.3.17)) or the copper/quinocofactor-containing amine oxidases. MAOs are found exclusively in the outer mitochondrial membrane of mammalian cells, and catalyze the deamination of primary, secondary, or tertiary amines. These flavoproteins have a firmly established role in the metabolism of aminergic neurotransmitters such as serotonin, norepinephrine, epinephrine, and dopamine [1]. Polyamine oxidases preferentially oxidize polyamines such as spermine and spermidine, and play a role in the regulation of cellular growth [2].

In contrast, the copper/quinocofactor-containing amine oxidases are active against only primary amines, and possess one of two quinone-containing cofactors: 2,4,5-trihydroxyphenylalanine quinone (topaquinone or TPQ) or lysyl tyrosylquinone (LTQ). Although the TPQ- and LTQ-containing amine oxidases both oxidatively deaminate primary amines through the use of an organic quinone-containing cofactor and a copper ion, these two enzyme families are non-homologous. LTQ is found in the lysyl oxidase (LOX)-like family of proteins (LOXL, E.C. 1.4.3.13), named for their ability to convert the ɛ-amino group of peptidyl lysine residues to an aldehyde. This peptidyl aldehyde product can condense with a neighboring lysine residue or react with a second lysine-derived aldehyde to form crosslinks important in the formation and stabilization of collagen and elastin [3]. Amine oxidases containing TPQ (CAOs for copper-containing amine oxidases, E.C. 1.4.3.21) were the first to be characterized in a growing number of bifunctional enzymes that contain “home-made” cofactors produced *in situ* by the post-translational modification of an endogenous amino acid side chain [4]. This review focuses on cofactor biogenesis and catalysis in the TPQ-containing CAOs, with a particular emphasis on the role X-ray crystallography has played in understanding these two processes.

## 2. Physiological Significance of CAOs

In the decades since the first discovery of amine oxidase activity in the blood plasma of sheep in 1929 [5], CAOs have been found to exist almost ubiquitously in aerobic organisms, taking on a myriad of functional roles depending on enzyme source, cellular location, and physiological substrate. In bacteria and yeast, CAOs are thought to play mainly a catabolic role, allowing for the use of primary amines as a sole carbon and/or nitrogen source for cellular growth [6,7].

In eukaryotes these enzymes have more poorly defined roles but are known to contribute to a variety of complex cellular activities. CAO activity helps regulate several processes in plants due to the compartment-specific production of product H_2_O_2_, which affects plant germination, seedling establishment, and root growth [8]. Hydrogen peroxide production is also associated with cell wall maturation and lignification during growth, as well as wound-healing and the reinforcement of cell walls during cellular stress due to pathogenic attack [9,10]. Moreover, amine catabolism is known to influence the plant stress response upon exposure to cadmium or excess salt [11].

Several CAO homologs are expressed in mammalian tissues. In order to study their distribution amongst mammalian species, four complete genes (*AOC1–4*, for “amine oxidase, copper-containing”) were cloned and characterized from porcine genomic DNA. All of these encode a bona fide CAO which displays catalytic activity toward primary amines [12]. These were used to identify orthologs in other mammalian genomes; all four *AOC* genes have been identified in cows, horses, dogs, mice, rats, chimpanzees, macaques, and humans [12]. *AOC1* encodes a soluble secretory enzyme known as diamine oxidase (DAO) [13], *AOC2* encodes a retina-specific amine oxidase [14], and *AOC3* encodes an endothelial vascular adhesion protein (known as VAP-1 in humans) [15]. *AOC4* was found to encode an additional serum/plasma CAO homologous to VAP-1, which lacks the *N*-terminal transmembrane domain found in VAP-1. However, the *AOC4* gene in humans is truncated and nonfunctional due to an internal stop codon, and *Aoc4* encoded by rodent genomes is present only in small fragments [12].

In mammals, nearly all biogenic primary amines can act as CAO substrates, including the neurotransmitters serotonin, norepinephrine, epinephrine, and dopamine. A large number of additional amines have been identified as CAO substrates, including methylamine, ethylamine, aminoacetone, benzylamine, phenylethylamine, agmatine, spermine, spermidine, histamine, tyramine, mescaline, putrescine, and cadaverine [16–18].

Members of the CAO protein family contribute to the regulation of a myriad of complex processes in mammals due to the diversity in their substrates, their wide distribution throughout mammalian tissues (including the brain, blood plasma, kidneys, placenta, and throughout the cardiovascular and gastrointestinal systems), and changes in expression and activity during disease and throughout pregnancy [19]. For example, histamine, a pro-inflammatory amine produced by mast cells upon allergic reaction or cellular damage, is cleared in human tissues by DAO [20]. Tumor cells are known to contain higher concentrations of amines than cells from normally proliferating tissues, and the oxidation of spermine or spermidine by CAOs produces acrolein, a known cytotoxin which can induce cellular death in cancerous tissues [21]. Agmatine (produced from the decarboxylation of arginine) binds to the α2 adrenergic receptor and imidazoline binding sites; its regulation through CAO activity has been shown to modulate withdrawal anxiety in rats [22].

Changes in CAO activity are correlated with a variety of human diseases, including diabetes mellitus, Alzheimer’s disease, and additional inflammatory disorders [23]. A truncated soluble form of membrane-bound VAP-1 known as semicarbazide-sensitive amine oxidase (SSAO) is known to mediate glucose uptake in adipocytes via the recruitment of the glucose transporter GLUT4 from vesicles within the cellular interior to the cell surface [24]. The soluble SSAO may also compensate for the absence of the *Aoc4* product in humans. VAP-1 also controls leukocyte extravasation to sites of inflammation through its activity against amine groups from solvent-exposed lysine residues of membrane-associated counter-receptors, including sialic acid binding Ig-like lectins (siglecs) [15,25]. Mice deficient in *Aoc3* expression exhibit impaired immune responses, but notably present no other detectable phenotypic changes [26]. The highly reactive aldehydes produced from the oxidation of methylamine and aminoacetone in human tissues contribute to protein cross-linking, β-amyloid aggregation, and advanced glycation end-product formation in patients with Alzheimer’s disease or diabetes mellitus [27,28]. With CAO activity playing a critical role in such numerous and diverse physiological processes, ranging from basic metabolism in bacteria and yeast to roles in multifaceted disease states in humans, an understanding of TPQ production and catalysis in these enzymes is critical to the development of any potential pharmaceutical therapies which may regulate their activity [23].

## 3. Post-Translationally Modified Amino Acid Cofactors

A large number of enzymes require low-molecular weight cofactors for catalytic activity, some of which are covalently attached to the protein component. Most cofactors are non-proteinaceous and independently synthesized before their association with the protein component of the relevant enzyme. However, it is now well-established that some enzymes utilize the post-translational modification of endogenous amino acid side chains to form cofactors *in situ* [4]. This approach enables an enzyme to avoid the energetic costs of cofactor import both to and within the protein, and furthermore may sequester potentially deleterious intermediates produced during cofactor formation. For example, TPQ as a free molecule is a known neurotoxin, however its production from an endogenous tyrosine residue in the deeply buried CAO active site circumvents this problem [29]. Most importantly, this strategy increases the diversity of chemical properties available within an enzyme active site beyond that afforded by the twenty canonical amino acids.

Variations in both the physical properties of these modified amino acid cofactors as well as the mechanisms employed for their formation are widespread. Many modifications involve the crosslinking of two amino acid side chains, for example cytochrome *c* oxidase and catalase (Tyr-His); tyrosinase, hemocyanin, and catechol oxidase (His-Cys); and galactose oxidase (Tyr-Cys) [30–36]. Cofactor biosynthesis can also involve the modification of a single side chain: sulfatases (cysteine converted to formylglycine); rubisco, urease, and phosphotriesterase (carbamylation of a lysine residue); NADH peroxidase, NADH oxidase, and nitrile hydratase (cysteine converted to cysteinesulfenic acid) [37–43].

**Figure 1:**
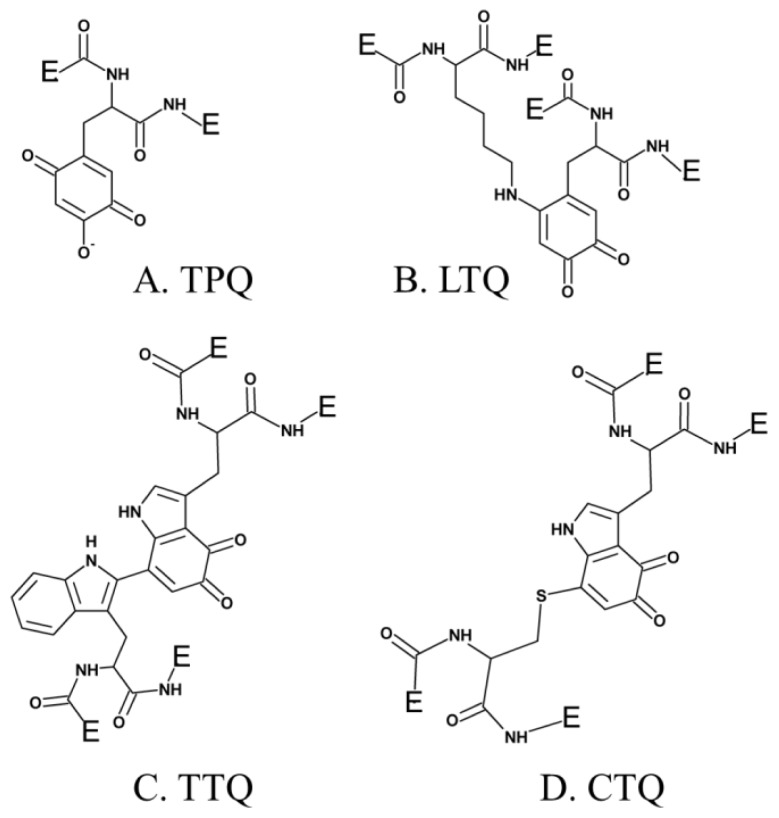
Quinone-containing cofactors formed by the post-translational modification of tyrosine or tryptophan side chains.

Several additional enzymes undergo post-translational modification of an endogenous tyrosine or tryptophan residue to form cofactors which contain a quinone moiety. To date, these include TPQ in CAO (Figure 1A) [29], LTQ in LOXL (Figure 1B) [44], tryptophan tryptophylquinone (TTQ) in bacterial amine dehydrogenase (Figure 1C) [45], and cysteine tryptophylquinone (CTQ) in bacterial quinohemoprotein amine dehydrogenase (Figure 1D) [46].

**Figure 2:**
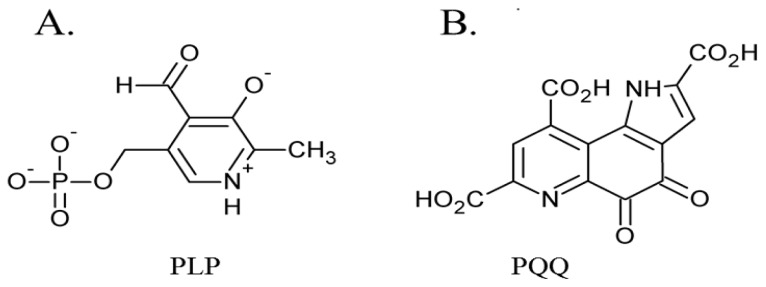
Structures of initially proposed CAO cofactors (**A**) PLP and (**B**) PQQ.

The first of these four quinocofactors to be identified was TPQ, which is located in the deeply-buried CAO active site. The formation of a chromophoric complex between CAO proteins and hydrazine derivatives had originally suggested pyridoxal phosphate (PLP) as a likely candidate for the CAO cofactor (Figure 2A) [47]. Two independent studies later reported the cofactor in *Bos taurus* serum amine oxidase (BSAO) to be covalently-bound pyrroloquinoline quinone (PQQ) [48,49] (Figure 2B), which was strengthened by resonance Raman experiments conclusively eliminating PLP as a possible CAO cofactor [50,51]. The true identity of the CAO cofactor was unequivocally confirmed to be topaquinone (Figure 1A) by Janes *et al.* through the use of mass spectrometry, ultraviolet-visible spectroscopy, and proton NMR studies of a pentapeptide isolated from proteolyzed BSAO in comparison to a peptide analog of 6-hydroxydopa [29].

## 4. Overall CAO Fold

The structures of CAOs isolated from a variety of organisms have been solved by X-ray crystallography (Table 1). Despite sharing only 20–40% sequence homology, they all adopt an archetypal fold which brings together a number of conserved residues to form the CAO active site where both cofactor biogenesis and catalysis take place. There are currently no crystal structures of LTQ-containing LOXL proteins, which are non-homologous to the TPQ-containing CAOs.

**Figure 3:**
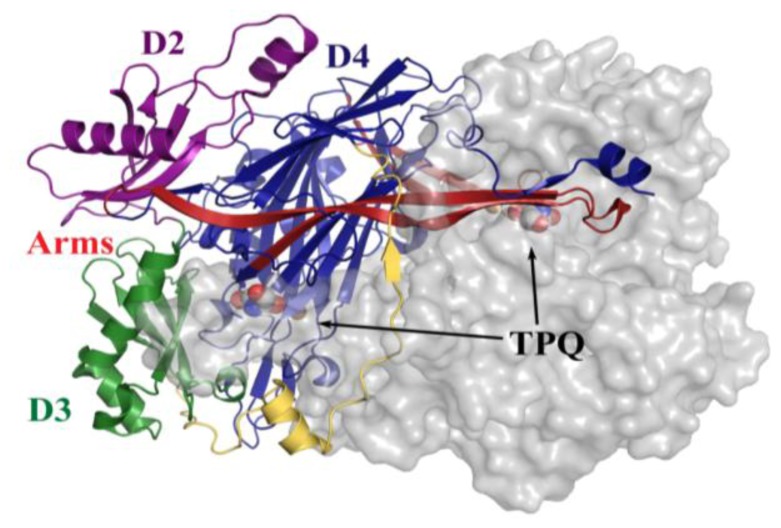
Overall fold of HPAO-1 viewed along the molecular dyad axis. One monomer is drawn in cartoon, and colored by domain (D2: purple; D3: green; D4: blue; connecting loop: yellow; β-hairpin arms: red). The second monomer is drawn as a semi-transparent molecular surface colored grey. TPQ from both monomers are drawn as space-filling spheres and colored by atom type (carbon, grey).

All CAOs structurally characterized thus far are homodimeric, with individual protein subunit masses ranging from ~70–80 kDa and eukaryotic CAOs having additional mass through glycosylation. The CAO monomer can be divided into four domains (D1–D4) organized along its primary sequence. The shape of the CAO dimer is similar to that of a mushroom, with the amino-terminal domain D1 (present only in the ECAO crystal structure) acting as a “stalk” (not present in HPAO-1, Figure 3) [59]. D2 and D3 are small α/β domains composed of ~100 residues each, and are thought to have arisen from a gene duplication event given their sequence homology and near identical topology (in HPAO-1, alignment of equivalent D2 and D3 main chain atoms gives a root-mean-square deviation (rmsd) of ~1.1 Å [55]) (purple (D2) and green (D3) in Figure 3).

The large carboxy-terminal domain D4 is composed of ~500 residues and contributes the majority of the conserved amino acids which form the enzyme active site (blue in Figure 3). The topology of D4 is that of a complex antiparallel β-sheet sandwich structure. Two β-hairpin arms protrude from the D4 domains of each monomer, which contain amino acid residues that interact with its partner in the protein dimer and form a portion of the back wall of the other monomer’s active site (red in Figure 3). These arms are thought to be important for the maintenance of dimer stability as well as the regulation of substrate specificity in CAOs. The region between monomers encloses a solvent-filled cavity known as the “inland lake,” and the dimer interface is extensive with a buried surface area comprising ~20% of the total surface area [58,59] (Figure 3).

All structurally characterized CAOs besides HPAO-1 and −2 contain two metal binding sites distinct from the active site copper, one of which is solvent exposed and one of which is less accessible (~36 Å and ~30 Å from the active site in ECAO, respectively) [62]. These metal sites primarily bind Ca(II), with the plant CAOs binding Ca(II) or Mn(II) at the less accessible site dependent on the availability of divalent metal ions [60,63]. The X-ray crystal structures of HPAO-1 and −2 indicate a salt bridge at the corresponding position (HPAO-1: Glu69 and Arg467, HPAO-2: Lys42 and Asp445) [55,56]. A study conducted using EDTA-treated ECAO found that calcium binding at these two sites is not essential for activity, but its removal decreases catalytic efficiency by ~60–90%, which can be partially recovered by the addition of an exogenous divalent cation [62]. This effect has been attributed to long-range structural changes which either alter the conformation of TPQ, or affect the dynamics of a hydrophobic channel important for oxygen transport to the active site.

## 5. Structure of the CAO Active Site

**Figure 4:**
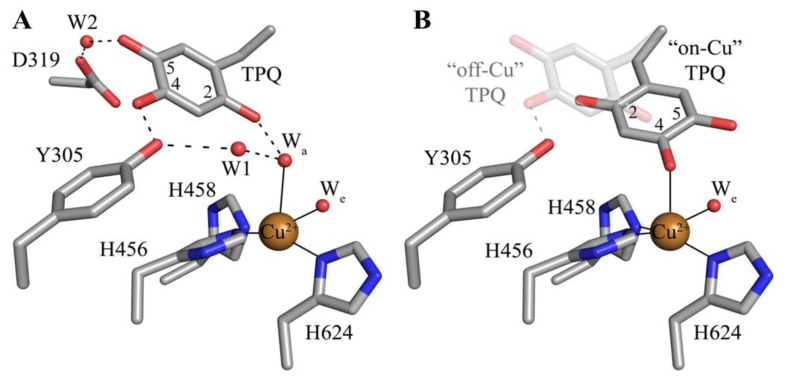
Crystal structures of the HPAO-1 active site (PDB code 2oov) with TPQ in an (**A**) “off-copper” or (**B**) “on-copper” conformation [55,64]. Residues are drawn in stick and colored by atom type (carbon, grey). Copper ions are drawn as gold spheres, and water molecules are drawn as small red spheres. Hydrogen bonds are indicated by dashed lines, and metal-ligand interactions are indicated by solid lines. In (**B**), the “off-copper” TPQ conformer from panel (**A**) is drawn in semi-transparent stick.

The deeply buried CAO active site contains a mononuclear type 2 Cu(II) ion bound in a distorted square pyramidal geometry by the imidazole groups of three conserved histidine residues at a distance of ~2.0 Å, a well-ordered axial water ligand at a distance of ~2.4 Å (W_a_), and a more labile equatorial water ligand visualized in some CAO structures at a distance of ~2.0 Å (W_e_) (Figure 4A). TPQ has been visualized in two conformations that are named based on the relationship between TPQ and the bound copper ion. The “off-copper” TPQ conformers do not act as copper ligands; instead, the O2 atom interacts with the metal center via the conserved axial water molecule (Figure 4A). The oxygen atom at position 4 of the TPQ ring is involved in a short hydrogen bond (~2.3 Å) with the phenolic hydroxyl of a conserved active site tyrosine residue (Tyr305 in HPAO-1). As this distance is less than 2.5 Å, it indicates a shared proton between Tyr305 and TPQ. The side chain of Tyr305 also interacts with the axial water ligand (W_a_) via an intervening water molecule (W1 in Figure 4A). The carbonyl group at position 5 of the TPQ ring points away from the copper center and toward the amine substrate channel. This off-copper species represents a catalytically productive CAO active site, as the C5 atom is the site of nucleophilic attack by amine substrates. A conserved aspartate residue, which acts as a general base during catalysis, sits near the substrate binding pocket (Asp319 in HPAO-1), with an intervening water molecule between it and the cofactor (W2 in Figure 4A). TPQ can also adopt an “on-copper” conformation, in which the ring has rotated about its Cα-Cβ and Cβ-Cγ bonds. In this conformation, TPQ displaces the axial water molecule which is coincident with the off-copper TPQ conformer (W_a_) and instead interacts directly with copper via its O4 atom. This represents an unproductive state because the C5 atom of TPQ is inaccessible for nucleophilic attack by substrate (Figure 4B).

## 6. Biogenesis of TPQ

**Scheme 1:**
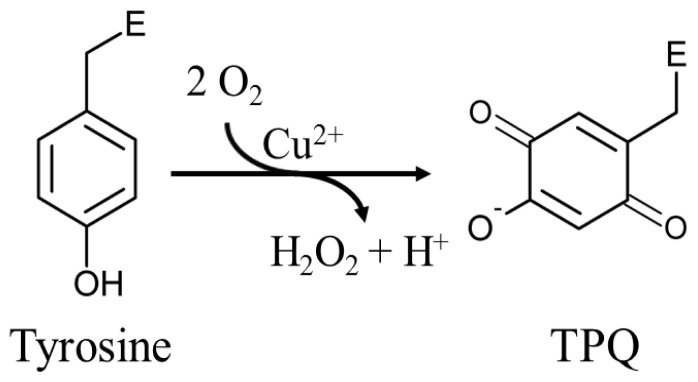
An endogenous tyrosine residue is converted to TPQ in an autocatalytic oxygen- and copper-dependent process. E represents the enzyme polypeptide.

The mechanism by which fully-folded CAOs synthesize a quinone-containing cofactor *in situ* has been an intriguing question in the field. A number of biochemical studies first established the process to be autocatalytic, requiring copper and oxygen but no auxiliary enzymatic activity or reducing equivalents [65,66]. Two moles of O_2_ are consumed for every mole of TPQ and H_2_O_2_ produced (Scheme 1) [67].

**Scheme 2:**
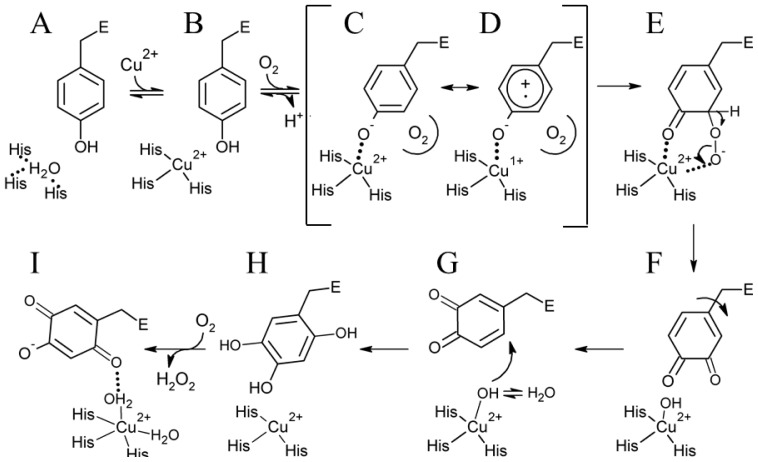
Proposed TPQ biogenesis mechanism in HPAO-1. Figure adapted from [70].

Kinetic and structural investigations using metal-free precursor (apo) protein from *Hansenula polymorpha* (apoHPAO-1) and *Arthrobacter globiformis* (apoAGAO), the only metal-free precursor CAO proteins currently available, have led to a proposed mechanism for TPQ biogenesis. This process in HPAO-1 is outlined in Scheme 2. ApoCAO is defined as amine oxidase protein which lacks both active site cofactors: a mononuclear copper ion and TPQ. The tyrosine residue which undergoes modification is contained within the conserved active site consensus sequence Thr-X-X-Asn-Tyr-Asp/Glu (precursor tyrosine residue underlined) [68]. TPQ biogenesis begins with apoCAO, in which the precursor tyrosine side chain is unmodified (A in Scheme 2) [58,69]. Copper binds at the active site, and is ligated by three strictly conserved histidine residues (A→B in Scheme 2). A kinetic study conducted using apoHPAO-1 with and without pre-bound copper reported a rate of TPQ formation which was unchanged with the pre-binding of Cu(II), indicating that copper binding is a fast process relative to the overall rate of biogenesis [70].

In HPAO-1, molecular oxygen then binds in a nearby off-copper hydrophobic pocket, which induces a conformational change in the precursor tyrosine side chain such that its hydroxyl group becomes oriented toward the copper (B→C in Scheme 2). Structural work suggests that at this point, the tyrosine residue is present in its protonated form, as indicated by the long distance between the phenolic oxygen and bound copper in crystal structures of anaerobic Cu(I)-apoHPAO-1 and Cu(II)-apoAGAO complexes (~2.8 Å and ~2.5 Å, respectively) [69,71].

Support for the oxygen-dependent formation of a tyrosine/copper complex comes from spectroscopic studies carried out using apoHPAO-1 [72]. The pre-incubation of apoHPAO-1 with copper followed by exposure to molecular oxygen resulted in a feature which absorbs at λ_max_ = 350 nm. This species decays isosbestically with the formation of TPQ, as indicated by a broad feature absorbing at 480 nm. Importantly, oxygen was found to be required but not consumed during this process, suggesting that oxygen binding results in a conformational change in the side chain of the precursor tyrosine residue. This movement, followed by deprotonation of the tyrosine, results in the formation of a ligand-metal charge transfer (LMCT) species which gives rise to the feature at 350 nm (C in Scheme 2). A putative oxygen binding pocket comprised of residues Met634, Tyr407, and Leu425 has been identified in HPAO-1, and the rate of biogenesis is linked to the volume of the hydrophobic residue at position 634 [73,74].

The Cu(II)-tyrosine complex, along with its Cu(I)-tyrosyl radical resonance form (D in Scheme 2), is activated for monooxygenation at position 3 of the tyrosine ring by the pre-bound O_2_, which forms a bridged peroxo adduct (C/D→E in Scheme 2). This species rapidly collapses to produce dopaquinone (DPQ) (E→F in Scheme 2). The DPQ ring flips ~180° about its Cα-Cβ bond by an unknown mechanism (F→G in Scheme 2), which leaves it positioned for the incorporation of a second oxygen atom at position 6 of the ring by a copper-activated water/hydroxyl (G→H in Scheme 2). This produces TPQ_red_ (2,4,5-trihydroxyphenylalanine = 2-electron reduced TPQ) (H in Scheme 2), which is then oxidized to mature TPQ with the concomitant reduction of molecular oxygen to hydrogen peroxide (H→I in Scheme 2). Lastly, TPQ moves to the catalytically productive off-copper position seen in structures of native CAO (I in Scheme 2).

**Figure 5:**
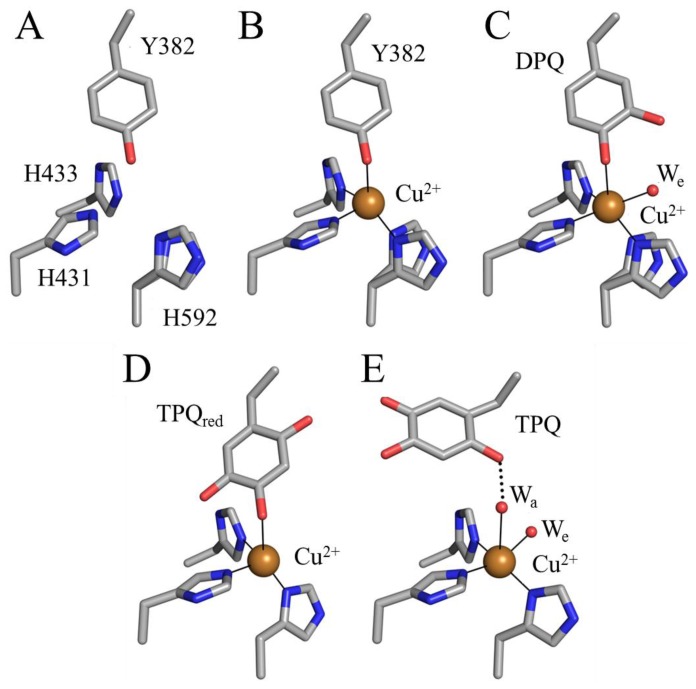
Structurally characterized TPQ biogenesis intermediates in AGAO [58,69]: (**A**) apoAGAO (PDB code 1avk); (**B**) apoAGAO/Cu(II) complex (PDB code 1ivu); (**C**) dopaquinone (DPQ)-containing early intermediate (PDB code 1ivv); (**D**) 2-electron reduced TPQ (TPQ_red_)-containing late intermediate (PDB code 1ivw) and (**E**) holoAGAO generated in the crystal (PDB code 1ivx). Residues are drawn in stick and colored by atom type (carbon, grey). Copper ions are drawn as gold spheres, and water molecules as small red spheres. Solid lines indicate metal-ligand interactions, and dashed lines indicate hydrogen bonds.

The preceding mechanism is strongly supported by a crystallographic investigation of TPQ biogenesis in apoAGAO [69]. The crystal structure of apoAGAO has been solved to a resolution of 2.2 Å, and unambiguously shows the precursor tyrosine residue (Y382 in AGAO) in its unmodified form with its hydroxyl group pointed toward the vacant metal binding site [58] (Figure 5A). This residue and the three conserved active site histidine residues that ligate copper in the native enzyme (His431, His433, and His592) are arranged around the empty metal binding site as if in a tetrahedral geometry. His431 and His433 adopt the conformers seen in the native enzyme, but His592 is present as two conformers, indicating some positional flexibility at this site. The two water molecules normally observed in the native CAO active site which ligate the copper (W_a_ and W_e_ in Figure 4A) are absent in the apoAGAO structure.

A series of crystal structures containing AGAO biogenesis intermediates were solved by exposing anaerobic apoAGAO crystals, which had been pre-bound with Cu(II), to O_2_ followed by freezing after different amounts of time [69]. This study produced structures of an anaerobic complex between apoAGAO and Cu(II) (flash-frozen after 0 min of O_2_ exposure), an early intermediate (flash-frozen after 10 min of O_2_ exposure), and a late intermediate (flash-frozen after 100 min of O_2_ exposure) formed during biogenesis (Figure 5B,C,D), which are consistent with the mechanism presented in Scheme 2. Finally, exposure of apoAGAO to O_2_ for a week before flash-freezing resulted in the structure of a species identical to holoAGAO, confirming that biogenesis can go to completion in the crystal (Figure 5E).

The structure of the anaerobic complex between apoAGAO and Cu(II) was solved to a resolution of 1.9 Å (Figure 5B). The electron density clearly shows the unmodified side chain of Tyr382, consistent with the requirement of molecular oxygen for TPQ biogenesis. The phenolic group of Tyr382 is thought to be protonated based on the distance between its hydroxyl group and the bound copper (~2.5 Å). Aside from the bound copper ion, this structure is essentially identical to that of apoAGAO (Figure 5A), and represents the first step of TPQ biogenesis, activating the precursor tyrosine side chain for monooxygenation at position 3 of the phenyl ring.

The structure of apoHPAO-1 in complex with Zn(II) (PDB code 1ekm) solved to 2.5 Å resolution in the presence of oxygen, reinforces the importance of the tyrosyl/copper complex in activating the precursor tyrosine ring for the initial oxygenation reaction [75]. Zinc is known to bind tightly at the CAO active site and resist displacement by copper [76]. Because zinc cannot support TPQ production, the structure of the Zn(II)-apoHPAO-1 complex mimics the copper bound species (B in Scheme 2). Similar to the anaerobic Cu(II)-apoAGAO complex, zinc was found to bind at the copper binding site and was ligated by the three conserved histidine residues and the unmodified precursor tyrosine residue in a tetrahedral geometry [75].

A crystal structure containing the dopaquinone intermediate formed during TPQ biogenesis (F in Scheme 2) was solved to a resolution of 2.1 Å (Figure 5C) [69]. The electron density confirmed that in this intermediate one oxygen atom had been inserted into the ring of Tyr382 to form dopaquinone. An additional feature of this structure is the presence of an equatorial water ligand at a distance ~2.1 Å from the copper center (W_e_ in Figure 5C) that is absent in the anaerobic complex between apoAGAO and Cu(II) (Figure 5B).

Freezing apoAGAO crystals after exposure to O_2_ for 100 minutes trapped TPQ_red_ (H in Scheme 2) solved to a resolution of 1.9 Å (Figure 5D) [69]. As indicated by the electron density, two oxygen atoms had been inserted into the Tyr382 ring, forming either TPQ_red_ or TPQ. This intermediate is formed only after rotation of the dopaquinone ring by ~180°, resulting in a species in which the O4 atom acts as a direct ligand to the bound copper. Single crystal microspectrophotometry experiments as well as the presence of a hydrogen bond between the O2 atom of the ring with the backbone carbonyl of Thr403 (indicating that the O2 atom is protonated), confirmed that this species is TPQ_red_ and not TPQ [69].

It was previously thought that only copper, the physiological relevant metal in CAOs, could support TPQ biogenesis. Studies conducted with apoHPAO-1 and apoAGAO, however, have revealed that biogenesis is supported by alternate metals *in vitro*, although at decreased rates [77,78]. In apoHPAO-1, biogenesis is supported by Cu(II), Cu(I), or Ni(II) bound at the active site, but does not occur upon Co(II) binding [78,79]. In contrast, biogenesis in apoAGAO is supported by Co(II) in addition to Cu(II) and Ni(II) (Cu(I) has not been tested) [77]. In both apoCAOs, Zn(II) binding at the active site renders the enzyme inert, as it does not support biogenesis and furthermore resists displacement by Cu(II) [75,77]. Several properties are thought to contribute to a metal’s ability to initiate and support biogenesis in apoCAOs. The low reduction potentials for the Ni(II)/Ni(I) and Co(II)/Co(I) couples disfavor a mechanism requiring the reduction of the metal for biogenesis [80,81]. Lewis acidity has been proposed to be important for biogenesis; however Zn(II), an effective Lewis acid, is unable to support TPQ synthesis, suggesting that reduction potential does play some role in determining which metals can initiate biogenesis [75,77].

**Figure 6:**
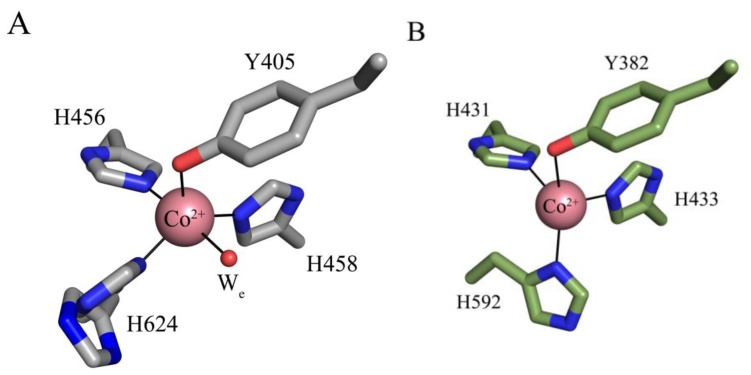
Active sites of (**A**) Co(II)-apoHPAO-1 (PDB code 3sxx) and (**B**) Co(II)-apoAGAO (PDB code 1wmp) [71,77]. Residues are drawn in stick and colored by atom type (apoHPAO1: carbon, grey; apoAGAO: carbon, green). Cobalt ions are drawn as pink spheres, and a water molecule as a small red sphere. Ligand-metal interactions are indicated by solid lines. Figure adapted from [71].

The crystal structures of apoHPAO-1, Cu(I)-apoHPAO-1 (prepared anaerobically) and Co(II)-apoHPAO-1 have also been reported [71]. The active sites of apoHPAO-1 and Cu(I)-apoHPAO-1 are nearly superimposable with those of apoAGAO and Cu(II)-apoAGAO, respectively [58,69]. The structure of Co(II)-apoHPAO-1, however, reveals that cobalt binds in apoHPAO-1 and apoAGAO with different geometries (Figure 6). In apoHPAO-1, the cobalt is 5-coordinate, bound in a distorted square pyramidal geometry by the imidazole groups of three histidine residues, the precursor tyrosine residue, and an equatorial water molecule (Figure 6A) [71]. In contrast, cobalt in the apoAGAO structure is bound tetrahedrally by the three conserved histidine residues and the precursor tyrosine residue (Figure 6B) [77]. Given that Co(II) supports biogenesis in apoAGAO but not apoHPAO-1, it is likely that differences in metal coordination influence the ability of Co(II) to support biogenesis.

**Figure 7:**
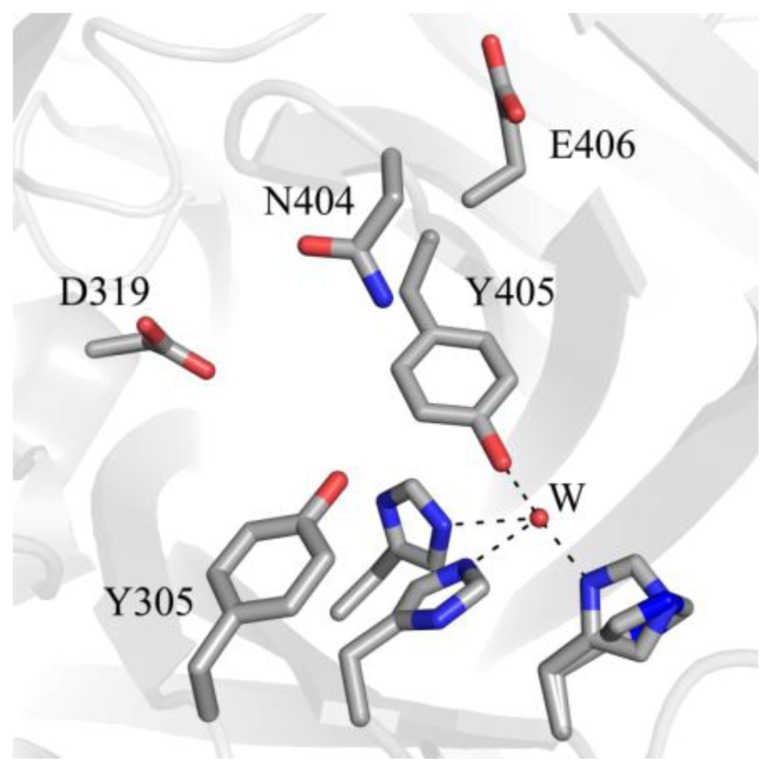
Amino acid residues in apoHPAO-1 shown to be involved in stabilizing biogenesis intermediates (PDB code 3sx1) [71]. Residues are drawn in stick and colored by atom type (carbon, grey). A water molecule is drawn as a small red sphere. Dashed lines indicate hydrogen bonds.

Biogenesis requires precise control in terms of positioning the precursor tyrosine residue and the intermediates formed during biogenesis within the active site. These species undergo rotations about their Cα-Cβ and Cβ-Cγ bonds to occupy both on- and off-copper positions. A number of active site residues, including some within the consensus sequence which contains the precursor tyrosine residue, contribute to this high level of conformational regulation by stabilizing the emerging cofactor in conformations accessed during biogenesis (Figure 7).

A strictly conserved active site tyrosine residue (Tyr305 in HPAO-1) is involved in a short hydrogen bond with the O4 atom of TPQ, helping to stabilize it in the appropriate position for catalysis of amine oxidation. The mutation of Tyr305 to a phenylalanine residue interferes with normal O–O bond cleavage during biogenesis and results in indiscriminant oxidative damage, indicating that this strictly conserved residue plays a role in conformational stabilization of the precursor tyrosine residue during biogenesis as well [82]. In HPAO-1, the mutation of the strictly conserved residue *N*-terminal to the precursor tyrosine (Asn404) to an alanine results in only 5–10% TPQ formation relative to the native enzyme, while the mutation of Asn404 to an aspartate results in a 2-fold decrease in *k*_TPQ_ [72,83]. The rate of TPQ biogenesis is also decreased by an order of magnitude when the residue *C*-terminal to the precursor tyrosine in HPAO-1 (Glu406) is mutated to a glutamine [72]. These effects illustrate the importance of the chemical properties of active site residues for biogenesis, such that mobility of the precursor tyrosine side chain is enabled during certain biogenesis steps, whereas a particular conformer is stabilized during others.

**Figure 8:**
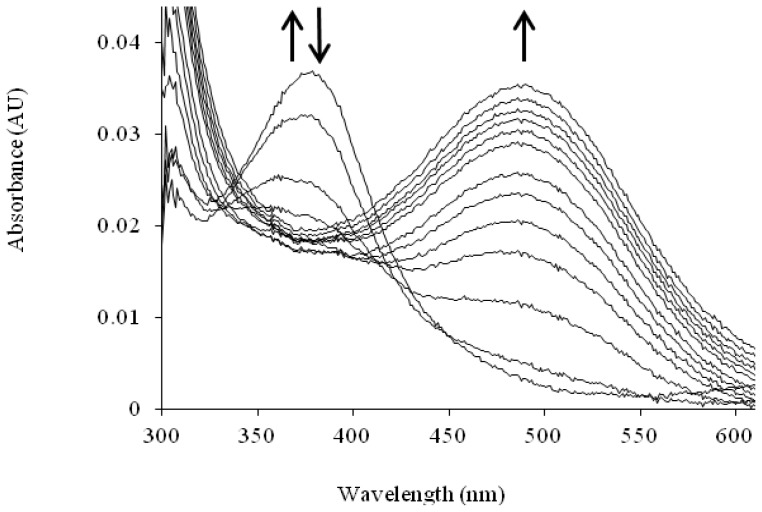
Solution UV/visible spectra showing the time course of the aerobic reconstitution of apoHPAO-1 with Cu(II) at pH 7.0. The directions of change for near-UV/visible absorbance features over time are indicated by arrows. Figure from [71].

The TPQ cofactor provides a chromophoric handle that enables changes in the electronic properties of TPQ to be monitored. ApoCAO protein is colorless with no near-UV/visible absorption peak, whereas the mature protein is yellow-pink in color (λ_max_ = 480 nm) due to electronic transitions within the cofactor [84]. When apoHPAO-1 is aerobically reconstituted with Cu(II), an intermediate absorbing at 380 nm forms and decays before the formation of the species at 480 nm which indicates mature TPQ (Figure 8). The rates of formation and decay of the 380 nm species are unaffected by pre-incubation with zinc or the addition of Cu(II) to the mature TPQ-containing HPAO-1. Thus, this species is thought to arise from an off-pathway LMCT interaction [72].

The copper bound at the CAO active site is ligated by three N and one/two O ligands and thus is type 2 or “non-blue” copper. Consequently, the intense spectral feature absorbing at ~600 nm associated with type 1 “blue” copper sites (ɛ = ~5000 M^−1^·cm^−1^) due to the presence of a cysteinic sulfur ligand is absent in CAOs [85]. The coupling of X-ray crystallography with single crystal spectroscopy is a powerful tool, allowing chromophoric species formed during enzymatic reactions to be identified before, during, and after X-ray data collection.

## 7. Catalysis in CAOs

**Scheme 3:**
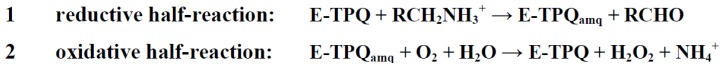
The catalytic half-reactions of CAOs. TPQ_amq_, aminoquinol. E, enzyme.

Catalysis in CAOs utilizes a ping-pong mechanism and can be thought of as two distinct half-reactions: (1) the oxidation of a primary amine substrate, generating product aldehyde and the 2-electron reduced aminoquinol form of the cofactor in which O5 is displaced by a substrate-derived amine group (known as the reductive half-reaction) and (2) the re-oxidation of TPQ with concomitant reduction of O_2_ to H_2_O_2_ and the release of NH_4_^+^ (known as the oxidative half-reaction) (Scheme 3).

**Scheme 4:**
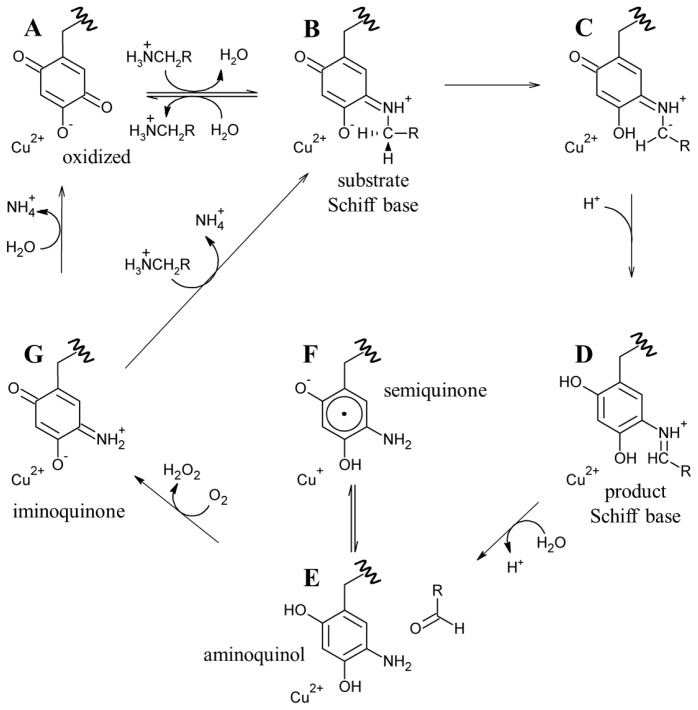
Proposed CAO catalytic mechanism. RCH_3_NH_3_^+^ is representative of all primary amine substrates.

The active sites of resting native CAOs contain oxidized TPQ and Cu(II) (A in Scheme 4). Catalysis is initiated through the nucleophilic attack by a primary amine on the C5 atom of TPQ forming a covalent substrate Schiff base complex (A→B in Scheme 4) [86]. Proton abstraction by a conserved aspartate residue from the C1 atom of the substrate, which is expected to have a decreased p*K*_a_, forms the corresponding product Schiff base species (D in Scheme 4) via the rapid rearrangement of a carbanionic intermediate (C→D in Scheme 4) [87]. Hydrolysis of the product Schiff base releases the corresponding aldehyde product and leaves the cofactor as a 2-electron reduced aminoquinol (D→E in Scheme 4) [88].

The aminoquinol represents the conclusion of the reductive half-reaction, and acts as the initial species in the oxidative half-reaction. While the study of CAOs from several sources has led to a general consensus regarding the reductive half-reaction, details concerning the oxidative half-reaction have remained unclear, particularly concerning the nature of the first electron transfer from reduced cofactor to O_2_ [89]. Reduced TPQ exists in an equilibrium between the aminoquinol/Cu(II) couple and a semiquinone radical/Cu(I) form (E and F, respectively, in Scheme 4) [90]. The distribution of the aminoquinol/semiquinone equilibrium is source-, pH-, and temperature-dependent [91–93]. At pH 7, CAOs derived from plant sources form as much as 40% semiquinone when anaerobically reduced with substrate, while other non-plant eukaryotic CAOs contain very low levels of semiquinone nearing the limit of detection [91]. Bacterial CAOs contain semiquinone at levels somewhere in between those of the two eukaryotic groups.

Two proposals to describe the first electron transfer to O_2_ have been put forth. The first utilizes an inner sphere electron transfer mechanism, in which O_2_ binds to the reduced copper in the semiquinone/Cu(I) couple. This is followed by electron transfer from Cu(I) to O_2_ to form copper(II)/superoxide. The transfer of another electron from the semiquinone and two protons to the superoxide yields the iminoquinone form of cofactor (G in Scheme 4) and hydrogen peroxide [94]. Support for an inner sphere electron transfer mechanism derives from the detection of the semiquinone radical form of TPQ [90] and the demonstration of a catalytically competent electron transfer rate from the aminoquinol to Cu(II) [93,95]. Additionally, azide, which is expected to ligate copper, was found to exhibit competitive inhibition with respect to O_2_ in PSAO and a CAO isolated from pig plasma, as well as partially competitive inhibition in DAO [96,97].

An outer sphere electron transfer mechanism has also been proposed, in which an electron from aminoquinol is transferred directly to O_2_ bound in a nearby hydrophobic pocket, forming superoxide and the semiquinone [98,99]. This mechanism does not involve an obligate change in the oxidation state of the copper, with its primary role being to stabilize the resultant superoxide. A kinetic study using BSAO demonstrated that the single electron reduction of O_2_ is rate-limiting during the oxidative half-reaction [98]. Chemical intuition suggests that this should be a fast process if the electron is derived from reduced Cu(I), thus an outer sphere electron transfer mechanism consistent with the kinetic data was proposed. Major support for an outer sphere electron transfer mechanism derives from work done with HPAO-1 [99,100]. The removal of copper from the HPAO-1 active site followed by reconstitution with Co(II) resulted in fully-functional protein with kinetic parameters under O_2_-saturating conditions indistinguishable from those of the native enzyme [100]. In addition, copper-depleted lentil seedling amine oxidase (LSAO) which was reconstituted with Co(II) regained partial catalytic competency [101]. The reduction potential of the Co(II)/Co(I) couple is very low, for example −400 to −500 mV *vs.* SHE in methionine synthase [80]. Co(II) is thus unlikely to be reduced during Co(II)-mediated catalysis, suggesting that a redox role for copper is unnecessary in the native enzyme. The use of a hydrophobic oxygen binding site near the copper is supported by kinetic data indicating that oxygen binding in HPAO-1 is noncompetitive with azide predicted to bind at the active site copper [102]. In addition, the oxidation of a model compound for the reduced cofactor has been shown to occur in the absence of metal [99]. Finally, the semiquinone intermediate in some anaerobically reduced CAOs is virtually undetectable, which lends additional support to an outer sphere electron transfer mechanism [98].

The issue of the first electron transfer from cofactor to O_2_ is being actively pursued, and it has been suggested that CAOs may be capable of using more than one mechanism to reduce O_2_ but that each different CAO has a clear preference for either inner or outer sphere electron transfer [89,103,104]. Regardless, O_2_ ultimately accepts two electrons and two protons from the cofactor, yielding H_2_O_2_ and the iminoquinone (G in Scheme 4). Hydrolysis of the iminoquinone releases product ammonium and regenerates oxidized TPQ (G→A in Scheme 4). Alternatively, when substrate levels are high the iminoquinone can react with a second amine substrate, releasing product ammonium and generating the substrate Schiff base (G→B in Scheme 4).

Catalysis in CAOs can be monitored spectroscopically owing to changes in the electronic form of TPQ, both in solution and *in crystallo* (Table 2).

**Figure 9:**
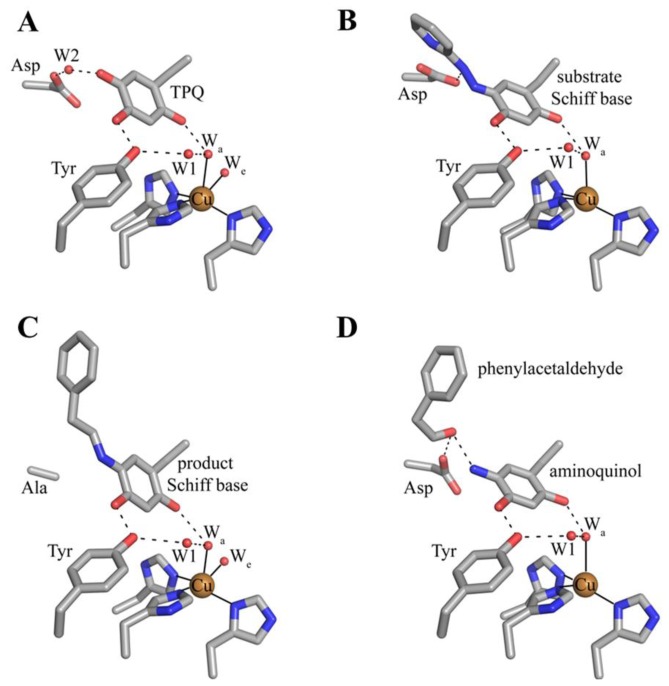
Structurally characterized intermediates formed during the CAO reductive half-reaction. (**A**) native HPAO-1 active site (PDB code 2oov) [64]; (**B**) ECAO containing a substrate Schiff base analog formed with the inhibitor 2-hydrazinopyridine (PDB code 1spu) [86]; (**C**) D298A AGAO containing product Schiff base (PDB code 2cwv) [113]; (**D**) substrate-reduced ECAO containing the aminoquinol (PDB code 1d6u) [88]. Residues are drawn in stick and colored by atom type (carbon, grey). Copper ions are drawn as gold spheres, and water molecules as small red spheres. Dashed lines indicate hydrogen bonds, and solid lines indicate metal-ligand interactions.

A number of catalytic intermediates formed during both half-reactions have been structurally characterized. The active sites from structures of native TPQ-containing CAOs from a variety of organisms (Table 1) are nearly identical, and contain oxidized TPQ, copper ligated by the imidazole groups of three strictly conserved histidine residues, an aspartate residue which acts as the catalytic base during catalysis, and several conserved water molecules. The active site structure of HPAO-1 is shown in Figure 9A [64]. As described previously, catalytically productive TPQ in the native enzyme is in an off-copper conformation with its C5 carbonyl pointed toward the substrate amine channel, ideally positioned for attack by a primary amine substrate. This conformation is stabilized by hydrogen bonding between the O2 atom of TPQ and an axial water molecule (W_a_ in Figure 9A), and between the O4 atom of TPQ and the hydroxyl of a conserved tyrosine residue. Results from resonance Raman spectroscopy using AGAO indicate that underivatized TPQ exhibits significant electron delocalization between the C2 and C4 oxygen atoms, with only the C5 atom possessing significant C=O character [111]. This is consistent with the formation of a covalent cofactor-substrate Schiff base complex following nucleophilic attack at the C5 position during catalysis as opposed to the C2 or C4 positions.

The structure of ECAO in a covalent complex with the inhibitor 2-hydrazinopyridine has been solved to a resolution of 2.0 Å [86] (Figure 9B). The inhibitor was found to bind to atom C5 of the cofactor, displacing the O5 atom and generating a Schiff base analog (B in Scheme 4). Because 2-hydrazinopyridine contains a nitrogen atom instead of a carbon at the C1 position, the covalent complex formed with the inhibitor cannot be deprotonated and thus accumulates in the crystal. The pyridine and quinone rings are not coplanar, suggesting that the complex is an analog of the substrate Schiff base and not the product Schiff base. A notable feature of this structure is the hydrogen bond between a nitrogen atom (corresponding to C1 in a physiological Schiff base) of the inhibitor/cofactor complex and an active site strictly conserved aspartate residue, which suggested this residue as the general catalytic base in the reductive half-reaction that abstracts a proton from the substrate Schiff base [86]. This was unequivocally confirmed through the study of twelve amino acid variants at this site in ECAO, which established that only glutamic acid had catalytic activity, although its *k*_cat_ was reduced by a factor of 6.4 × 10^4^ compared to the native aspartic acid [86,87]. Recently, AGAO has also been solved in complex with three different hydrazine inhibitors: benzylhydrazine, 4-hydroxybenzylhydrazine, and phenylhydrazine [112]. As in ECAO, these bind as hydrazone adducts analogous to substrate Schiff base.

The mutation of the active site aspartate residue (D298) to an alanine in AGAO results in a decrease in catalytic efficiency by ~10^6^ orders of magnitude, with the low level of activity possibly due to water acting as a base [113]. The incubation of D298A AGAO crystals with the physiological substrate 2-phenylethylamine for one week before freeze-trapping and structure solution resulted in a 1.85 Å resolution crystal structure containing a product Schiff base intermediate (Figure 9C). This assignment was confirmed by single crystal microspectrophotometry [113]. The product Schiff base has not been observed in solution, presumably due to the fast rate of hydrolysis which produces aldehyde and the aminoquinol (D→E in Scheme 4). In the crystal, however, the reaction is considerably slower and the product Schiff base is able to accumulate. The C2 atom of the product is coplanar with the TPQ ring and the imine double bond in this structure, which is anticipated for the product Schiff base formed with 2-phenylethylamine (Figure 9C).

The 2-electron reduced aminoquinol is the final cofactor intermediate formed during the reductive half-reaction, and marks the initiation of the oxidative half-reaction. Crystals containing the aminoquinol at the active site were obtained by freeze-trapping crystals of ECAO after their anaerobic reduction with 2-phenylethylamine. The structure of this species was solved to a resolution of 2.4 Å (Figure 9D) [88]. Single crystal microspectrophotometry indicated that the species was bleached, which is consistent with the aminoquinol (Table 2) [106]. Present in the off-copper conformation, the aminoquinol interacts with the copper ion through the axial water molecule seen in native CAO structures. A surprising feature in this structure was the presence of phenylacetaldehyde product bound at the back of the active site. Crystal contacts appeared to inhibit interdomain movement such that aldehyde remained trapped in the enzyme at the back of the active site [88].

**Figure 10:**
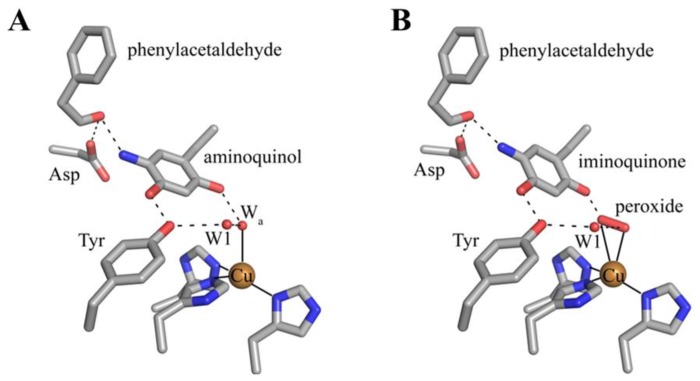
Structurally characterized intermediates formed during the CAO oxidative half-reaction. (**A**) Substrate reduced ECAO containing the aminoquinol (PDB code 1d6u) [88]. (**B**) ECAO containing the iminoquinone (PDB code 1d6z) [88]. Residues are drawn in stick and colored by atom type (carbon, grey). Copper ions are drawn as gold spheres, and water molecules as small red spheres. Hydrogen bonding interactions are indicated by dashed lines, and metal-ligand interactions are indicated by solid lines.

Intermediates formed during the oxidative half-reaction which have been structurally characterized are shown in Figure 10. The oxidative half-reaction involves the reoxidation of TPQ, and begins with TPQ in an equilibrium between the aminoquinol/Cu(II) couple and a semiquinone radical/Cu(I) species. A structure containing the aminoquinol has been solved, and is described in the preceding paragraph. Currently there is no published crystal structure of a reduced CAO containing semiquinone and Cu(I).

A structure of the iminoquinone intermediate formed during catalysis was solved after the prolonged aerobic exposure of an ECAO crystal to 2-phenylethylamine (Figure 10B) [88]. This steady-state structure contains not only the iminoquinone, but also an oxygen species which has displaced the axial water molecule in the structure containing aminoquinol (Figure 10A). Phenylacetaldehyde product remains bound in the back of the active site in the same position it occupies in the aminoquinol-containing structure (Figure 10A). The side-on geometry of the oxygen species in the presence of iminoquinone and product aldehyde suggested that this was most likely the product hydrogen peroxide. The presence of an oxygen species bound at the copper coincident with product aldehyde demonstrates that in the crystal, the ping-pong kinetics seen in solution have been disrupted.

Finally, it is well documented that CAOs function as obligate dimers, and evidence suggests that some, but not all, CAOs exhibit cooperativity through long-range conformational changes propagated through the CAO dimer from one active site to the other [86,114–118]. BSAO and ANAO exhibit half-site reactivity with regard to hydrazine inhibitors [114,115,118]. In addition, kinetic work using HPAO-1 heterodimers which contain zinc bound in one active site of the HPAO-1 dimer and copper in the second, could not efficiently carry out the oxidative half-reaction at either active site, suggesting that communication between the two metal binding sites influences oxidative chemistry [119]. It has been suggested that metal binding in one CAO active site induces conformational changes through a network of interactions that crosses the dimer interface [119].

## 8. CAO Channels and Access to the Active Site

**Figure 11:**
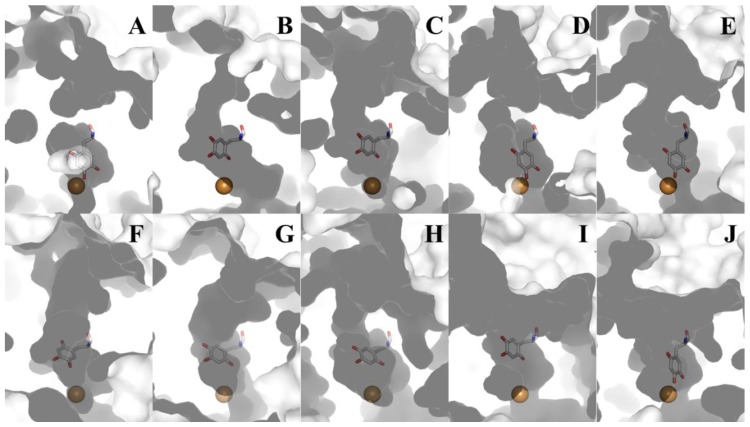
Cross-section through the amine substrate channels of different CAOs. Each enzyme is drawn as a molecular surface. TPQ is drawn in stick and colored by atom type (carbon, white). Copper ions are drawn as gold spheres. (**A**) ECAO; (**B**) HPAO-1; (**C**) HPAO-2; (**D**) VAP-1; (**E**) DAO; (**F**) PSAO; (**G**) AGAO; (**H**) BSAO; (**I**) ANAO; (**J**) PPLO (Table 1).

The substrates consumed during CAO catalysis (a primary amine and O_2_) take different paths to the same deeply-buried active site. A distinct substrate amine channel leads from the enzyme surface to the C5 atom of TPQ, the site of nucleophilic attack during catalysis (a distance of ~18 Å in HPAO-1) (Figure 11B). Despite the well-conserved structural homology of the overall CAO fold, the dimensions and shape of the amine substrate channel vary significantly depending on enzyme source. Amongst CAO homologs, only four amino acids within this channel display any sequence homology (besides the invariant aspartate residue which acts as the catalytic base and the consensus sequence containing the precursor tyrosine residue which is converted to TPQ). Position 323 (HPAO-1 numbering) corresponds to an aromatic residue, position 155 corresponds to either a proline or serine residue, position 305 corresponds to a tyrosine residue important in stabilizing TPQ species and off-copper conformations, and position 156 corresponds to a bulky hydrophobic residue. This channel not only allows substrate amine access to the site of catalytic turnover, but also functions as an egress for product aldehyde exiting the active site.

Substrate preference in different CAOs is also determined by the chemical properties of the residues lining the amine substrate/aldehyde product channel, as well as the general shape and size of the channel. These vary significantly depending on enzyme source, with channels ranging from nearly obstructed (ECAO, Figure 11A) to channels so broad that entire peptides can be accommodated and serve as substrates ((I) ANAO and (J) PPLO, Figure 11).

An additional proposed role for the residues lining the amine substrate channel involves the stereospecificity of the proton abstraction step during the CAO reductive half-reaction. The stereospecificity varies depending on enzyme source and the identity of the amine substrate used [86,120–122]. For example, PSAO selectively abstracts the *pro-S* proton from dopamine, tyramine, and benzylamine during the reductive half-reaction, while BSAO is non-stereoselective when dopamine or tyramine are used as a substrate, but abstracts the *pro-S* proton from Schiff base complexes formed with benzylamine, *p*-hydroxybenzylamine, and 3-methylbutylamine [120,121]. In contrast, AGAO is known to selectively abstract the *pro-S* proton from Schiff base complexes formed with all substrates tested thus far [122]. In all CAO homologs, the *pro-S* proton from the Schiff base species formed with benzylamine is abstracted in favor of the *pro-R* proton. Given the strong structural homology between CAO active site residues, it has been suggested that the conformation of the substrate Schiff base complex itself is more important than the positions of active site residues relative to the amine substrate in determining the stereospecificity of the proton abstraction step [123]. The residues lining the amine substrate channel are important for the accommodation of Schiff base intermediates and display relatively low sequence homology amongst CAOs compared to other regions of the protein. The interactions between these residues and the Schiff base complexes consequently influence the stereospecificity of the proton abstraction step during catalysis in a species- and/or substrate-dependent manner.

The second substrate consumed during catalysis, molecular oxygen, had previously been thought to travel to the CAO active site via the “inland lake” where the two monomers in the CAO dimer meet. While it is possible that the inland lake may act as a reservoir for molecular oxygen (based on potential of mean force (PMF) maps which illustrate regions of low free energy for the placement of O_2_ inside the protein matrix) it has been proposed that the narrow polar channel connecting this region to the active site is more appropriate for exiting H_2_O_2_ [64]. This is consistent with both the short length and polar nature of H_2_O_2_.

In order to visualize molecular oxygen movement through the protein matrix, crystal structures of the complex between CAO and xenon have been solved from several sources, including PSAO [124], PPLO [124], ECAO [125], AGAO [124], and HPAO-1 [64]. Xenon has the same volume as molecular oxygen and mimics oxygen binding in hydrophobic pockets. Several recurrent O_2_ binding sites have been identified from these xenon complexes, including in the β-sheet sandwich fold of the catalytic D4 domain, the amine substrate channel, and a channel from the inland lake.

**Figure 12:**
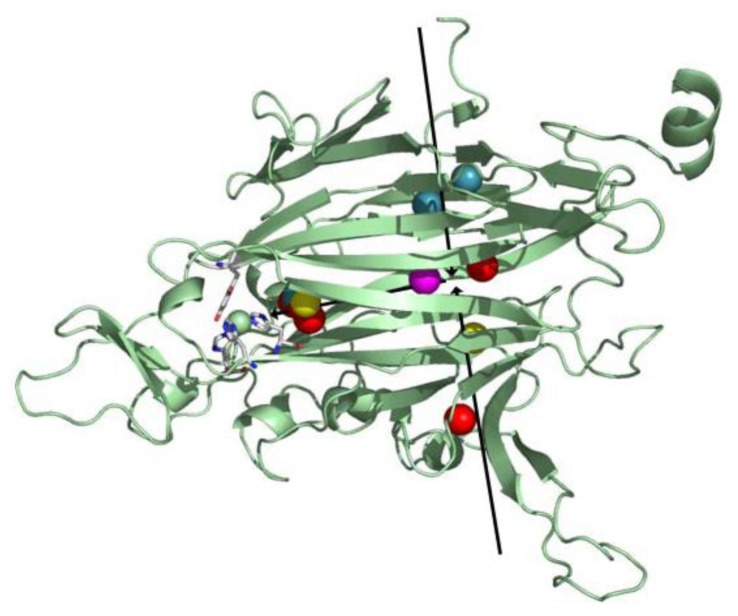
Overlay of xenon binding sites in xenon/CAO complexes. Domain D4 from one HPAO-1 monomer is drawn as a green cartoon, and active site residues are depicted in stick and colored by atom type (carbon, white). Xenon atoms are drawn as spheres and colored by enzyme source (PSAO, red; PPLO, yellow; AGAO, blue; HPAO-1, magenta). Arrows indicate the direction of O_2_ movement toward the active site. Figure from [64].

Taken together, crystallographic and PMF data from these studies suggest several species-dependent O_2_ points of entry into the protein interior (two are shown as black arrows in Figure 12). After its initial entry into the protein matrix, O_2_ is transiently held within the hydrophobic interior of domain D4, followed by migration closer to the active site where it is activated (Figure 12). Though the interior of domain D4 is largely composed of hydrophobic residues in all CAO homologs, source-specific differences in the primary amino acid sequence of D4 could account for the different points of entry utilized by O_2_ in different CAO homologs.

## 9. Summary

The studies on CAOs demonstrate the power and complementarity of X-ray crystallography in understanding catalytic processes when combined with kinetic analysis, mutagenesis and spectroscopy, both in solution and in the crystal. The crystalline environment can dramatically slow down the rates of catalytic steps that depend on enzyme dynamics, and even alter the catalytic mechanism, as seen in the loss of ping-pong kinetics in ECAO catalysis *in crystallo*. However, when coupled to complementary single crystal spectroscopy any structural changes can effectively be equated to catalytic intermediates observed spectroscopically in solution. X-ray crystallography has been, and will remain, an important tool in the quest for understanding both CAO biogenesis and catalysis at the molecular level.

## Figures and Tables

**Table 1:** TPQ-containing CAOs with available X-ray crystal structures.

Source	Organism	Reference
Mammalian	*Homo sapiens*	VAP-1/SSAO [52,53]; DAO [20];
	*Bos taurus*	BSAO [54]
Yeast	*Hansenula polymorpha*	HPAO-1 [55]; HPAO-2 [56]
	*Pichia pastoris*	PPLO [57]
Bacterial	*Arthrobacter globiformis*	AGAO [58]
	*Escherichia coli*	ECAO [59]
Plant	*Pisum savitum*	PSAO [60]
Fungal	*Aspergillus nidulans*	ANAO [61]

**Table 2:** λ_max_ of spectroscopic intermediates formed during CAO catalysis.

Catalytic Intermediate	λ_max_
TPQ (quinone) [105]	480
Substrate Schiff base [106]	340
Product Schiff base [83,107,108]	380
Aminoquinol [106]	310
Semiquinone [90]	360, 435, 465
Iminoquinone [109,110]	450 or 350 (if charge is delocalized, as in HPAO-1)
Cu(II)-peroxy [109]	410

## Abbreviations

AGAO: *Arthrobacter globiformis* amine oxidase
ANAO: *Aspergillus nidulans* amine oxidase
apoCAO: precursor form of copper amine oxidase
BSAO: bovine serum amine oxidase
CAO: copper amine oxidase
CTQ: cysteine tryptophylquinone
DAO: diamine oxidase
DPQ: dopaquinone
ECAO: *Escherichia coli* amine oxidase
HPAO-1: *Hansenula polymorpha* amine oxidase that prefers small aliphatic primary amines
HPAO-2: *Hansenula polymorpha* amine oxidase that prefers aromatic primary amines
LMCT: ligand-metal charge transfer
LOX: lysyl oxidase
LOXL: lysyl oxidase-like proteins
LSAO: lentil seedling amine oxidase
LTQ: lysyl tyrosylquinone
MAO: monoamine oxidase
PDB: Protein Data Bank
PLP: pyridoxal phosphate
PMF: potential of mean force
PPLO: *Pichia pastoris* lysyl oxidase
PQQ: pyrroloquinoline quinone
PSAO: *Pisum sativum* amine oxidase
rmsd: root-mean-square deviation
SSAO: semicarbazide-sensitive amine oxidase
topaquinone or TPQ: 2,4,5-trihydroxyphenylalanine quinone
aminoquinol or TPQ_amq_: 2,4-dihydroxy-5-aminophenylalanine
TPQ_red_: 2,4,5-trihydroxyphenylalanine
TTQ: tryptophan tryptophylquinone
VAP-1: vascular adhesion protein-1
